# Relationship between response to first-line steroid treatment in adult immune thrombocytopenic purpura and the course of the disease

**DOI:** 10.1016/j.htct.2024.03.008

**Published:** 2024-07-23

**Authors:** Esra Seçkin, Rafiye Ciftciler

**Affiliations:** a Selcuk University Faculty of Medicine, Department of Internal Medicine, Konya, Turkey; b Selcuk University, Faculty of Medicine, Department of Hematology, Konya, Turkey

**Keywords:** Immune thrombocytopenic purpura, Thrombocytopenia, Bleeding, Steroid

## Abstract

**Background:**

Immune thrombocytopenic purpura, a recurrent autoimmune disease, is characterized by thrombocytopenia, purpura and hemorrhagic episodes with the main factor in the pathogenesis of this disease being autoantibodies against platelets. Since the 1950s, first-line treatment has been glucocorticoids that have indirect and direct effects on thrombocytopenia. Although the characteristics associated with the chronicization of immune thrombocytopenic purpura at the time of diagnosis have been investigated in previous studies, no study was found in the literature investigating the relationship between the response to first-line steroid treatment and the course of the disease, the aim of this study.

**Materials and methods:**

This retrospective, single center study revisited electronic files of patients with a diagnosis of immune thrombocytopenic purpura between September 2012 and September at the Department of Clinical Hematology, Selcuk University Faculty of Medicine 2022. The platelet count had been confirmed by peripheral blood smears of patients with a platelet count ≤30 × 10^9^/L. The bleeding status of patients at the time of diagnosis was evaluated according to the immune thrombocytopenic purpura bleeding score. Patient responses to treatment were categorized in three groups: a platelet count ≤30 × 10^9^/L was defined as no-response, a platelet count of 30–100 × 10^9^/L was defined as partial response, and a platelet count >100 × 10^9^/L was defined as complete response. Subsequently, patients in the partial or complete response groups were divided into two subgroups: patients who remained in remission for less than or more than six months.

**Results:**

A total of 100 patients were included in the study; 73 % were in the young (19–65 years old) and 27 % in the old (>65 years old) age group. Most of the patients were female (69 %). Forty-one patients were hospitalized without bleeding. The complete response rate to first-line treatment was 61 %. There was no significant difference between the agents given in first-line treatment in terms of response and length of remission.

**Conclusion:**

The main purpose of immune thrombocytopenic purpura treatment is to prevent severe bleeding rather than bringing the platelet count to normal values. Glucocorticoids, the first step of treatment, provide high response rates. There is no significant difference between glucocorticoid agents in terms of response to treatment and long-term remission. The points to be considered in the selection of glucocorticoid agents are the side effect profiles, ease of administration and individualization of treatment.

## Introduction

Immune thrombocytopenic purpura (ITP) is an acquired condition of thrombocytopenia caused by autoantibodies against platelet antigens.^1^ Two mechanisms are active in the pathogenesis of ITP. The first is a decrease in platelet production however, the main causative mechanism is a decrease in platelet count due to antiplatelet antibodies.^2^

ITP used to be called idiopathic thrombocytopenic purpura in older sources however, today it is called ITP. Its etiology may include infections such as *H. pylori* and human immunodeficiency virus (HIV), malignancies such as lymphomas and other autoimmune-related diseases. In these diseases, antiplatelet antibodies cause platelet destruction.

Since ITP is frequently a chronic illness in adults, the prevalence much outweighs the incidence. Prevalence was about 8/100,000 in children and 12/100,000 in adults according to an assessment in the United States.^3^ The overall incidence was 2.9/100,000 person-years in a database review from the French National Health Insurance System, which was restricted to ITP cases requiring hospitalization and/or chronic therapy. The incidence peaked in over 60-year-old individuals reaching 9/100,000 person-years in over 75-year-old men.^4^

ITP was included in the medical literature in 1025 thanks to Ibn Sina. In 1735, a German doctor Paul Gottlieb Werlof defined skin and mucosal bleeding that developed after an infection as “morbus maculosus haemorrhagicus”.^5^ In 1905, Marino argued that thrombocytopenia could occur through immune mechanisms.^5^^,^^6^ ITP is an autoimmune disease characterized by low platelet count, bleeding events and purpura nevertheless most patients are diagnosed with asymptomatic thrombocytopenia in random blood tests. For symptomatic patients, this condition is primarily associated with bleeding due to thrombocytopenia. In addition to bleeding symptoms, fatigue and low quality of life are also observed.^7^

When the platelet count is found to be <100 × 10^9^/L in blood tests, it should be confirmed by a peripheral smear. ITP is a diagnosis of exclusion in patients with isolated thrombocytopenia with the most important component of the diagnostic evaluation being the exclusion of other causes.

The primary goal of treatment is to prevent clinically significant bleeding and to treat bleeding if it is present. When drawing up a treatment plan for the patient, it is first necessary to decide whether treatment is indicated and determine the urgency of treatment. The next steps vary according to the persistence of thrombocytopenia, the treatment given and the response to treatment.^8^^,^^9^ The first-line treatment in ITP has long been glucocorticoids^5^ which decrease autoantibody formation and prevent phagocytosis of platelets coated with autoantibodies via the mononuclear phagocytic system.^10^ The response rate to first-line treatment is 70–90 % however, the preservation rate of this response in patients who respond to treatment is only 15–20 %.^11^ Information on parameters that predict the course of ITP in patients is not sufficient and so determination of these parameters is very important to ensure that ITP treatment is specific to each individual. If these parameters are determined, the course of the disease can be predicted, thus unnecessary drug use and possible side effects can be avoided.

The main aim of the current study was to determine the relationship between the response to first-line steroid treatment in adult ITP and the course of the disease. In addition, other aims were to evaluate and compare clinical features and laboratory results at the time of diagnosis.

## Materials and methods

### Study design and data collection

The records of patients with a diagnosis of ITP who were followed up at the Department of Clinical Hematology, Selcuk University Faculty of Medicine between September 2012 and September 2022 were revisited and 100 patients who met the inclusion criteria were included in the study. A peripheral smear confirmed platelet counts ≤30 × 10^9^/L. The bleeding status of the patients at the time of diagnosis was evaluated according to the ITP bleeding score.^12^ This score comprises 11 site-specific grades from 0 (none) to 2 (marked bleeding) assessed at nine anatomical sites by history over the previous week. In addition, two of these sites, skin and oral, are assessed by physical examination (PE). The ‘worst ever’ bleeding experienced at each site is graded using the same system.^12^ The demographic characteristics of the patients, their medical histories, laboratory test results performed at diagnosis were recorded. Moreover, data on the first-line treatment received, response to treatment, whether the disease recurred and, if so, the time of recurrence, whether second-line treatment was received, and if second-line treatment was received, the treatments they received were noted in detail. Patient responses to treatment were categorized in three groups: a platelet count <30 × 10^9^/L was defined as no-response, from 30 to 100 × 10^9^/L was defined as partial response, and >100 × 10^9^/L was defined as complete response. Subsequently, patients in the partial or complete response groups were divided into two subgroups: patients who remained in remission for less than six months and those who remained in remission for more than six months. Over 18-year-old patients whose first-line treatment for ITP was initiated at the clinic of the study were included. Under 18-year-old patients, women who were pregnant when thrombocytopenia was detected, and patients whose primary care for ITP was started in another center were excluded.

### Statistical analyses

Statistical analyses utilized the Statistical Package for Social Sciences (SPSS) version 25 (IBM, Armonk, NY, USA). Analytic techniques (Kolmogorov-Smirnov/Shapiro-Wilk test) and visual approaches (histograms, probability plots) were used to ascertain whether the variables were normally distributed or not. The chi-square test was used to make statistical comparisons of categorical data. Student's *t*-test was employed to compare continuous numerical data for two independent samples. The effect profile of parameters on binary results and their relationships with the result were identified using logistic regression analysis. The relationships between quantitative parameters and categorical parameters are summarized by schematizing with boxplot graphs. Spearman correlation analysis was used for correlation relationships of quantitative parameters. A type 1 (α) error of 5 % was accepted thus significance was set for *p*-values <0.05.

## Results

Of the patients included in this study, 69 were women and 31 were men. The relationships of gender in respect to age, laboratory results and categorical data were investigated. Creatinine (*p*-value <0.001) and hemoglobin (Hb) (*p*-value <0.001) levels were found to be lower in women compared to men.

The relationships of age groups in relation to laboratory results, recurrence time (months), and bleeding stages were also assessed. White blood cell (WBC) (p-value = 0.01) and Hb (*p*-value = 0.02) values were higher in the 19–65 age group compared to the >65 age group, while creatinine (*p*-value = 0.03) and lactate dehydrogenase (LDH - *p*-value = 0.02) values were higher in the >65 age group.

No statistically significant association was observed in respect to bleeding in terms of gender, age group, patient history, first-line treatment agent and treatment response, or second-line treatment response.

No statistical relationship was observed of quantitative and categorical parameters with the first-line treatment agent, treatment response, or length of remission. Post-treatment platelet levels were highest in the group with >6 months remission and lowest in the group without remission (*p*-value <0.001). On the other hand, recurrence time in months (*p*-value <0.001) and Hb (*p*-value = 0.03) values were highest in the group with >6 months remission and lowest in the group with <6 months remission. When analyzed according to the bleeding phases, no significant association was observed between the bleeding phases and the groups (Table 1).

**Table 1:** Analysis of the relationship of first-line treatment modalities and outcomes with quantitative and categorical data.

	**Treatment modalities**			**Response to first-line treatment**				**remission period after the first-line treatment**			
	Methylprednisolone (*n* = 74)	Dexamethasone (*n* = 26)	p-value	No response to treatment (*n* = 21)	Partial Response (*n* = 18)	Complete Response (*n* = 61)	p-value	≤6 months (*n* = 18)	>6 months (*n* = 61)	No Remission (*n* = 21)	p-value
Median (min-max											
Platelets - at diagnosis (x 10^9^/L)	9.7 (2.0–38.7)	13.0 (2.0–30.0)	0.15^a^	10.0 (2.0–29.0)	16.0 (4.0–38.7)	10.0 (2.0–38.0)	0.49^c^	10.5 (3.0–23.0)	12.0 (2.0–38.7)	10.0 (2.0–29.0)	0.91 ^c^
WBC (x 10^9^/L)	7.71 (1.18–26.0)	7.4 (1.8–18.6)	0.81 a	8.0 (2.5–23.0)	6.9 (1.8–11.4)	7.7 (1.18–26.0)	0.62 ^c^	6.95 (1.8–18.6)	7.7 (1.18–26.0)	8.0 (2.5–23.0)	0.70 ^c^
ALT (U/L)	19 (8–133)	17 (7–124)	0.67 a	23 (9–88)	16.5 (7–60)	18 (8–133)	0.0503 ^c^	17 (7–124)	18 (8–133)	23 (9–88)	0.54 ^c^
AST (U/L)	20 (8–102)	22.5 (14–55)	0.55 a	22 (12–60)	19.5 (10–66)	21 (8–102)	0.20 ^c^	22 (13–57)	20 (8–102)	22 (12–60)	0.64 ^c^
Creatinine (mg/dL)	0.71 (0.39–1.5)	0.74 (0.5–1.41)	0.36 a	0.65 (0.49–0.95)	0.8 (0.48–1.16)	0.73 (0.39–1.5)	0.45 ^c^	0.67 (0.39–1.18)	0.75 (0.48–1.5)	0.65 (0.49–0.95)	0.13 ^c^
LDH (U/L)	256 (139–880)	262 (158–432)	0.72 a	264 (149–880)	245 (139–487)	257 (163–564)	0.66 ^c^	286 (180–442)	257 (139–564)	264 (149–880)	0.68 ^c^
Platelet - post-treatment) (x 10^9^/L)	162.5 (3.0–589.0)	123.0 (7.0–622.0)	0.15 a	24.0 (3.0–578.0)	71.0 (38.6–269.0)	197.0 (106–622)	0.12 ^c^	143.5 (40.0–622.0)	170 (38.6–589.0)	24.0 (3.0–578.0)	**<0.001**^c^
Recurrence time (months)	5 (1–66)	5 (1–12)	0.34 a	5 (1–36)	5 (1–12)	5 (1–66)	0.91 ^c^	2 (1–6)	16 (9–66)	5 (1–36)	**<0.001**^c^
Mean ± SD											
Age (years)	52.91 ± 16.72	51.58 ± 16.69	0.74^b^	48.57 ± 16.40	55.28 ± 13.97	53.13 ± 18.68	0.45 ^d^	52.39 ± 20.35	53.98 ± 16.96	48.57 ± 16.40	0.47^d^
Hb (g/dL)	12.61 ± 2.64	13.03 ± 2.38	0.47 ^b^	12.38 ± 2.18	12.60 ± 2.78	12.87 ± 2.66	0.73 ^d^	11.44 ± 2.96	13.21 ± 2.46	12.38 ± 2.18	**0.03**^d^
n: (%)											
Gender											
Female	50 (72.5)	19 (27.5)	0.60 *	16 (23.2)	11 (15.9)	42 (60.9)	0.59 *	13 (18.8)	40 (58.0)	16 (23.2)	0.62 *
Male	24 (77.4)	7 (22.6)		5 (16.1)	7 (22.6)	19 (61.3)		5 (16.1)	21 (67.8)	5 (16.1)	
Age Group											
19–65 years	53 (72.6)	20 (27.4)	0.60*	17 (23.3)	14 (19.2)	42 (57.5)	0.49 *	13 (17.8)	43 (58.9)	17 (23.3)	0.65 *
>65 years	21 (77.8)	6 (22.2)		4 (14.8)	4 (14.8)	19 (70.4)		5 (18.5)	18 (66.7)	4 (14.8)	
Bleeding Phase											
Phase 0	27 (65.9)	14 (34.1)	0.20 *	11 (26.8)	10 (24.4)	20 (48.8)	0.0503 ⁎⁎	8 (19.5)	22 (53.7)	11 (26.8)	0.07⁎⁎
Phase 1	28 (75.7)	9 (24.3)		3 (8.1)	6 (16.2)	28 (75.7)		9 (24.3)	25 (67.6)	3 (8.1)	
Phase 2	19 (86.4)	3 (13.6)		7 (31.8)	2 (9.1)	13 (59.1)		1 (4.5)	14 (63.6)	7 (31.8)	

ALT: Alkaline phosphatase; AST: Aspartate phosphatase; LDH: Lactate dehydrogenase; WBC: White blood cells; Hb: Hemoglobin Min: Minimum; Max: Maximum; SD: Standard deviation.

Parameters that show normal distribution are expressed as means ± SD, and those that do not show normal distribution are expressed as medians (min-max) (IQR).

a Mann-Whitney U test ^b^ Independent *t*-test ^c^Kruskal-Wallis Test ^d^ANOVA test.

⁎ Pearson chi-square analysis.

⁎⁎ Fisher's exact test The bleeding score comprised 11 grades from 0 (none) to 2 (marked bleeding) assessed at nine anatomical sites by history over the previous week (Hx). In addition, two of these sites, skin and oral, were also assessed by physical examination (PE). The ‘worst ever’ bleeding experienced at each site was graded using the same system.^12^

There was no statistically significant association between the agents used in first-line treatment (methylprednisolone and dexamethasone) and length of remission (*p*-value = 0.39 - Table 2).

**Table 2:** Relationship and distribution characteristics between remission status and first-line treatment agents.

Remission duration	Methylprednisolone n (%)	Dexamethasone n (%)	Total n (%)	χ^2^	p-value*
≤6 months	11 (61.1)	7 (38.9)	18 (100)	1.901	0.39
>6 months	47 (77.0)	14 (23.0)	61 (100)		
None	16 (76.2)	5 (23.8)	21 (100)		
Total	74 (74.0)	26 (26.0)	100 (100)		

⁎ Pearson chi-square.

No statistically significant association was found between response to first-line treatment and length of remission (*p*-value = 0.54 - Table 3). Moreover, no statistical association was found between First- and second-line treatment agents and response/length of remission (Table 4).

**Table 3:** Relationship between first-line treatment response and remission.

Response	Remission ≤6 months - n (%)	Remission >6 months - n (%)	Total	p-value
Complete (Platelet >100 × 10^9^/L)	13 (21.3)	48 (78.7)	61 (100)	0.54*
Partial (Platelet 30–100 × 10^9^/L)	5 (27.8)	13 (72.2)	18 (100)	
Total	18 (22.8)	61 (77.2)	79 (100)	

⁎ Fisher's exact test.

**Table 4:** Statistical relationships of first- and second-line treatment agents with treatment responses and length of remission.

		First-line treatment agent		p-value
		Methylprednisolone (*n* = 74) n (%)	Dexamethasone (*n* = 26) n (%)	
Response to Treatment	None	16 (21.6)	5 (19.2)	0.14 *
	Partial	10 (13.5)	8 (30.8)	
	Complete	48 (64.9)	13 (50)	
Remission	≤6 months	11 (14.9)	7 (26.9)	0.38 *
	>6 months	47 (63.5)	14 (53.8)	
	None	16 (21.6)	5 (19.3)	
		**Second-line treatment agent**		
		**Eltrombopag** (*n* = 13) n (%)	**Other Treatments** (*n* = 9) n (%) †	p-value
Response to Treatment	None	4 (30.8)	3 (33.3)	0.99 ⁎⁎
	Partial	2 (15.4)	2 (22.3)	
	Complete	7 (53.8)	4 (44.4)	
Remission	≤6 months	4 (30.8)	2 (22.2)	0.87 ⁎⁎
	>6 months	4 (30.8)	4 (44.4)	
	None	5 (38.4)	3 (33.4)	

† Rituximab (*n* = 7), Mycophenolate (*n* = 1), Splenectomy (*n* = 1).

⁎ Pearson chi-square test ** Fisher's exact test.

Thirty-eight (38 %) patients experienced recurrence; recurrence occurred most frequently in the fifth (*n* = 7; 7 %) and second (*n* = 6; 6 %) months. No statistical association was found between recurrence and gender, age group, medical history, first-line and second-line treatment agents, first-line treatment responses and bleeding status. A close relationship was observed between Hb levels and recurrence with low Hb levels being found to have an effect profile on recurrence (*p*-value = 0.047).

## Discussion

On examining the literature, many studies examining the predictive features of ITP at the time of diagnosis are identified.^13^^,^^14^ However, no large-scale study examining the relationship between the response to first-line treatment and the course of the disease was found in the literature; therefore, this study is unique. Experienced clinicians consider ITP to be a disease that affects young women with some studies confirming this thesis.^15^ In this study, there were more people in the younger age group (19–65 - 73 %) than in the older group (>65 - 27 %). In the current study, the mean age was 52.3 ± 17.77 and the female to male ratio was 69:31. Thus, this study shows that ITP is more common in young female patients and even so gender and age do not affect the bleeding status.

Unless ITP occurs secondary to another disease, WBC and Hb values are typically normal.^16^ The median WBC was 8.39 × 10^3^/µL and the median Hb was 12.72 ± 2.57 g/dL. These values are compatible with the literature; the WBC and Hb values were higher in the 19–65 age group. The reason that Hb values were lower in elderly patients may be due to the increase in comorbidities.

According to studies, these patients generally go to hospital without symptoms however some patients go with varying severities of bleeding in different parts of the body. These bleedings most commonly occur in the skin or mucosa^17^ but are generally not life-threatening. In this study, consistent with the literature, 41 % of the patients went to the clinic without bleeding or symptoms. The most common bleedings were mostly mild of the skin, the nasal mucosa, and oral mucosa with life-threatening bleeding being seen in only 2 % of cases. ITP should definitely be one of the first preliminary diagnoses to be considered in cases of bleeding of the skin and mucosa or in thrombocytopenia detected incidentally by blood tests.

Although the bleeding seen in ITP is mild, it should be kept in mind that life-threatening bleeding is possible. Although bleeding tends to be higher in patients with more severe thrombocytopenia^18^ many studies have reported that the correlation between platelet count and bleeding severity is weak.^19^ In the present study, consistent with this information, the platelet count at the time of diagnosis was found to be significantly lower in the group of patients with bleeding. However, again in accordance with the literature, no correlation was found between platelet count and bleeding severity. This situation observed in ITP may be due to the fact that the existing circulating platelets are younger and have more hemostatic activity.

Glucocorticoids are used as the standard first-line treatment of ITP.^20^ In a study conducted by Oluç et al. using methylprednisolone treatment, 57.4 % of the patients showed complete response and 20.2 % showed partial response.^21^ In this study, 61 % of complete response and 18 % of partial response were observed in patients who received first-line treatment. These results are consistent with the literature.

In a study including 1138 patients with ITP, the patients were divided into methylprednisolone and dexamethasone groups according to the treatment agent they received. Although the response to dexamethasone was faster than the response to methylprednisolone, the overall response rates were similar.^22^ In the current study, the complete response rate was 64.9 % in the group receiving methylprednisolone and 50 % in the group receiving dexamethasone without statistically significant differences between the treatments in terms of response. In other words, the agent chosen in first-line treatment does not change the response. The important points to consider when choosing an agent are the side effect profiles of the given agent, ease of administration and individualization of the treatment.

In a study conducted in 2016, the length of remission was compared between methylprednisolone and dexamethasone treatment. The ≥6 month remission rate was found to be 59 % in the methylprednisolone group and 79 % in the dexamethasone group.^22^ In the present study, the ≥6 month remission rate was 63.5 % for the methylprednisolone group and 53.8 % for the dexamethasone group without any statistically significant difference between treatment responses and length of remission.

Different studies have been conducted about the long-term course of ITP. In these studies, the rates of remission vary between 49 % and 67 %.^23^ In this study recurrence was observed in 38 % of the patients, most frequently in the fifth (7 %) and second months (6 %). There was no statistically significant association between recurrence and response to treatment or between recurrence and age, gender and comorbidities. However, significantly lower Hb values were in the group with recurrence, which may be due to iron deficiency anemia caused by chronic bleeding.

## Conclusion

In conclusion, response to glucocorticoids does not affect the duration of remission and relapses in ITP patients. Additionally, there is no significant difference between the glucocorticoid agents in terms of response to treatment and length of remission. The points to be considered in the selection of glucocorticoid agents are the side effects of the agents, ease of administration, and individualization of treatment. In cases where there is no response to first-line treatment or in cases of recurrence, second-line treatments with different mechanisms of action can be used.

## Role of the funding source

None.

## Informed consent statement

Informed consent for the procedure was obtained from blood donors for this study. The Selcuk University Ethics Committee approved this study (2022/430–01.11.2022).

## Conflicts of interest

The authors of this paper have no conflict of interests, including specific financial interests, relationships, and/or affiliations relevant to the subject matter or materials included.
